# Growth Conditions and Cell Cycle Phase Modulate Phase Transition Temperatures in RBL-2H3 Derived Plasma Membrane Vesicles

**DOI:** 10.1371/journal.pone.0137741

**Published:** 2015-09-14

**Authors:** Erin M. Gray, Gladys Díaz-Vázquez, Sarah L. Veatch

**Affiliations:** Department of Biophysics, University of Michigan, Ann Arbor, Michigan, United States of America; University of Colorado Boulder, UNITED STATES

## Abstract

Giant plasma membrane vesicle (GPMV) isolated from a flask of RBL-2H3 cells appear uniform at physiological temperatures and contain coexisting liquid-ordered and liquid-disordered phases at low temperatures. While a single GPMV transitions between these two states at a well-defined temperature, there is significant vesicle-to-vesicle heterogeneity in a single preparation of cells, and average transition temperatures can vary significantly between preparations. In this study, we explore how GPMV transition temperatures depend on growth conditions, and find that average transition temperatures are negatively correlated with average cell density over 15°C in transition temperature and nearly three orders of magnitude in average surface density. In addition, average transition temperatures are reduced by close to 10°C when GPMVs are isolated from cells starved of serum overnight, and elevated transition temperatures are restored when serum-starved cells are incubated in serum-containing media for 12h. We also investigated variation in transition temperature of GPMVs isolated from cells synchronized at the G1/S border through a double Thymidine block and find that average transition temperatures are systematically higher in GPMVs produced from G1 or M phase cells than in GPMVs prepared from S or G1 phase cells. Reduced miscibility transition temperatures are also observed in GPMVs prepared from cells treated with TRAIL to induce apoptosis or sphingomyelinase, and in some cases a gel phase is observed at temperatures above the miscibility transition in these vesicles. We conclude that at least some variability in GPMV transition temperature arises from variation in the local density of cells and asynchrony of the cell cycle. It is hypothesized that GPMV transition temperatures are a proxy for the magnitude of lipid-mediated membrane heterogeneity in intact cell plasma membranes at growth temperatures. If so, these results suggest that cells tune their plasma membrane composition in order to control the magnitude of membrane heterogeneity in response to different growth conditions.

## Introduction

Giant plasma membrane vesicles (GPMVs) isolated from cortical cytoskeleton are a powerful model system for probing some properties of the cell surface. These vesicles are easily isolated from living cells through several distinct chemical treatments [1–3], contain a broad array of plasma membrane proteins and lipids [4,5], and their physical properties can be easily probed using a variety of experimental methods widely used to study purified model membranes including fluorescence microscopy. GPMVs undergo a miscibility phase transition below cellular growth temperature, under which vesicles contain coexisting liquid-ordered (L_o_) and liquid-disordered (L_d_) phases that are visible using fluorescent probes sensitive to composition or membrane ordering [6–9]. Depending on the isolation protocol, GPMV transition temperatures vary between close to 0°C and up to roughly 30°C [9], and significant vesicle-to-vesicle and day-to-day variation in transition temperatures are found even when the same isolation protocol is used [10]. The main goal of this work is to investigate possible sources of this heterogeneity.

Even though cells in culture are frequently clonal, cells can exhibit variability in membrane composition when grown at different densities or with different nutrient levels. Previous studies have demonstrated that cells arrested in G0 or G1 through serum starvation or contact inhibition have altered plasma membrane lipid composition [11,12] with reduced sphingomyelin content and increased diacylglycerol and ceramide levels, both conditions expected to modulate miscibility transition temperatures in purified model membranes. Another source of GPMV transition temperature heterogeneity could arise from cells being unsynchronized within the cell cycle, since there are well documented changes in lipid composition at different cell cycle positions [12–15]. Plasma membrane composition is also altered in apoptosis, such as when sphingomyelin lipids are converted to ceramides at early stages of this pathway [16]. Replacement of sphingomyelin lipids with ceramides have well documented effects on miscibility transition temperatures in model membranes [17–19], again suggesting that GPMVs from apoptotic cells will have altered transition temperatures. The vast majority of past work characterizing plasma membrane lipids within the cell cycle has focused on the important roles of lipids as second messengers, although a few studies have noted changes in the mobility of plasma membrane lipids and proteins as a function of cell cycle position [20,21] and changes in the phase behavior of total lipid extracts [15] suggesting that membrane physical properties are also affected.

In this study, we have characterized how growth conditions and cell cycle position alter miscibility transition temperatures of isolated GPMVs. We find that transition temperatures are highest when GPMVs are prepared from sparsely plated cells and lowest when prepared from cells plated in confluent monolayers or when cells are starved of serum. Systematic variation in GPMV transition temperatures is also observed when GPMVs are prepared from synchronized cells as they progress through the cell cycle, and transition temperatures are reduced in GPMVs isolated from cells undergoing apoptosis. Overall we conclude that variation in the surface density of cells is a major contributor to heterogeneity of GPMV transition temperatures, and speculate that systematic changes in transition temperatures provide evidence for biological tuning of this phase transition in intact cells.

## Materials and Methods

### Cells and reagents

RBL-2H3 cells [22] were maintained in MEM media with 20% FBS and 0.1% Gentamycin at 37°C in 5% CO_2_. Cells were provided by Barbara Baird and David Holowka (Cornell University, Ithaca NY). Unless otherwise specified, cells were prepared by first detaching cells by incubation with trypsin-EDTA (0.25%, Gibco) for 5min at 37°C, followed by seeding cells in plastic dishes or flasks. Freshly seeded samples were incubated in complete media for 18-24h at 37°C prior to GPMV isolation. Unless otherwise specified, all reagents were purchased from Sigma Aldrich (St. Louis, MO).

### GPMV preparation and imaging

GPMVs were prepared through incubation with low concentrations of dithiothreitol (DTT, 2mM) and formaldehyde (25mM) in the presence of calcium (2mM) for 1h as described previously [1,6,10,23]. Prior to GPMV formation, cells were labeled with DiI-C_12_ (Life Technologies, Carlsbad, CA; 2μg/ml in 1% methanol) for 10min. GPMVs were imaged on an inverted microscope (IX81; Olympus, Center Valley, PA) with a 40x air objective (0.95 NA), epi-illumination using an Hg lamp and Cy3 filter set (Chroma Technology, Bellows Falls, VT). Temperature was controlled using a home built peltier stage described previously [23,24] coupled to a PID controller (Oven Industries, Mechanicsburg, PA), and images were recorded using a SCMOS camera (Neo; Andor, South Windsor, CT).

### GPMV transition temperature determination

GPMV transition temperatures were measured as described previously [23]. Briefly, images were acquired of fields of GPMVs over a range of temperatures such that at least 100 vesicles could be identified at each temperature. After imaging, individual vesicles were identified as having a single liquid phase, two coexisting liquid phases, or containing some gel phase in the case of the sphingomyelinase treatment measurements. This information was compiled into a plot showing the percentage of vesicles with two liquid phases as a function of temperature, which was fit to a sigmoid function to determine the temperature where 50% of vesicles contained two coexisting liquid phases (T_misc_)

$$\%seperated=100\times \left(1-\frac{1}{1+e^{-(T-Tmisc)/B}}\right),$$ (1)

where B is a parameter describing the width of the transition. Errors in single measurements of T_misc_ (σT_misc_) are 68% confidence interval estimates of the parameter estimate directly from the fit. Error bounds for a transition temperature shift (ΔT_misc_) are given by $\sqrt{\sigma_{C}{}^{2}+\sigma_{M}{}^{2}}$ where σ_C_ is the error in measuring T_misc_ of the untreated control and σ_M_ is the error in measuring T_misc_ of the treated sample. When multiple measurements are included in a single reported value of T_misc_, a weighted average is performed. Weights for the i^th^ measurement is given by $w=\frac{1}{\sigma_{i}{}^{2}}{\left(\sum \frac{1}{\sigma_{i}{}^{2}}\right)}^{-1}$, where σ_i_ is the error in T_misc_ for the individual measurement. The error bounds on the resulting average are given by $\sigma_{avg}={\left(\sum \frac{1}{\sigma_{i}{}^{2}}\right)}^{-1/2}$.

### Quantification of cell surface density

After labeling with DiI-C_12_, fields of cells were imaged using a 20X air objective. After imaging, cell centers were identified in images using a simple automated imaging processing algorithm which identified local maxima in band-pass filtered images followed by manual identification of cells missed by the algorithm. Reported errors represent the variation observed between fields of cells in the same sample.

### Preparation of cells at different densities within the same dish

RBL cells were suspended as described above to 2 million cells/ml and 5 spots of 10μl each were deposited onto a 10cm^2^ plastic petri dish (for a total of 100,000 cells). This was incubated for 1h to allow for cell attachment, then an additional 100,000 cells were added in 2ml of media and dishes were incubated overnight at 37°C. This resulted in circular areas of high cell density surrounded by sparse cells.

### Serum depletion measurements

Cells grown under typical growth conditions were suspended with trypsin then incubated with media containing either normal (20%) or no (0%) serum concentrations. Different numbers of cells were plated in each serum condition to account for differing growth rates under low serum conditions (5x10^5^ cells in normal serum vs. 7.5x10^5^ for serum depleted in a 25cm^2^ flask). For serum add-back measurements, cells incubated in 0% serum overnight, then 0% serum media was replaced with media containing 20% serum. GPMVs were prepared from cells starting at the specified times after media replacement.

### Cell synchronization measurements

Cells were synchronized through a double thymidine block [25]. RBL-2H3 cells were grown to 25%-30% confluence, then suspended in media containing 2mM thymidine and grown for 18h (1^st^ block). Cells were then suspended with trypsin-EDTA (Life Technologies), divided into multiple containers, and allowed to grow in normal growth media for 9h before again incubating for 17h with media containing 2mM thymidine (2^nd^ block). Cells were released from the 2^nd^ block by again incubating with growth media without added thymidine and GPMVs were prepared starting at the indicated time-points.

### TRAIL and sphingomyelinase measurements

Cells grown under normal conditions were suspended, counted, and plated into several containers with the cell numbers (typically 5x10^5^ cells in a 10cm^2^ dish or 1x10^6^ cells in a 25cm^2^ flask). Prior to GPMV preparation, treated cells were either incubated with 100ng/ml TRAIL (Fitzgerald Industries, Acton, MA) or 1.7μg/ml (0.32U/ml) purified sphingomyelinase from Staphylococcus aureus (Sigma) for 30min at 37°C in growth media. An additional container of cells was left untreated as a control. GPMVs from treated and control dishes were then prepared as described above.

### Preparation and imaging of giant unilamellar vesicles (GUVs)

GUVs were prepared through electroformation and imaged as described previously [26]. Vesicles contained 33% Dioleoylphosphocholine (DOPC), 33% Cholesterol (Chol), and either 33% Brain sphingomyelin (BSM) or a combination of BSM Brain Ceramide (Bcer) adding to a total of 33%. All lipids were purchased from Avanti Polar Lipids (Birmingham, AL). Vesicles also contained 0.8% Texas Red dipalmitoyl-phosphatidylethanolamine (TR-DPPE, Molecular Probes, Eugene, OR). Vesicles were imaged by epifluorescence microscopy the experimental set-up and temperature controlled stage described previously [26].

### Two dimensional Ising model simulations

2D Ising model simulations with a conserved order parameter were conducted using standard Monte Carlo algorithms in the presence of a weak applied magnetic field as described previously [24,27]. Simulations were conducted on 200 by 200 pixel square lattice with periodic boundary conditions and updates were performed using non-local spin flips to decrease equilibration times. A weak magnetic field was applied favoring white pixels within a circular region with diameter of 45 pixels placed at the center of the simulation box. The magnitude of the applied field was set at 1% of the enthalpy between neighboring spins of the same type for all pixels within the circular region. Time-average images are constructed from 2000 simulation snap-shots.

## Results

### The miscibility transition is heterogeneous in a single preparation of GPMVs

**Fig 1:**
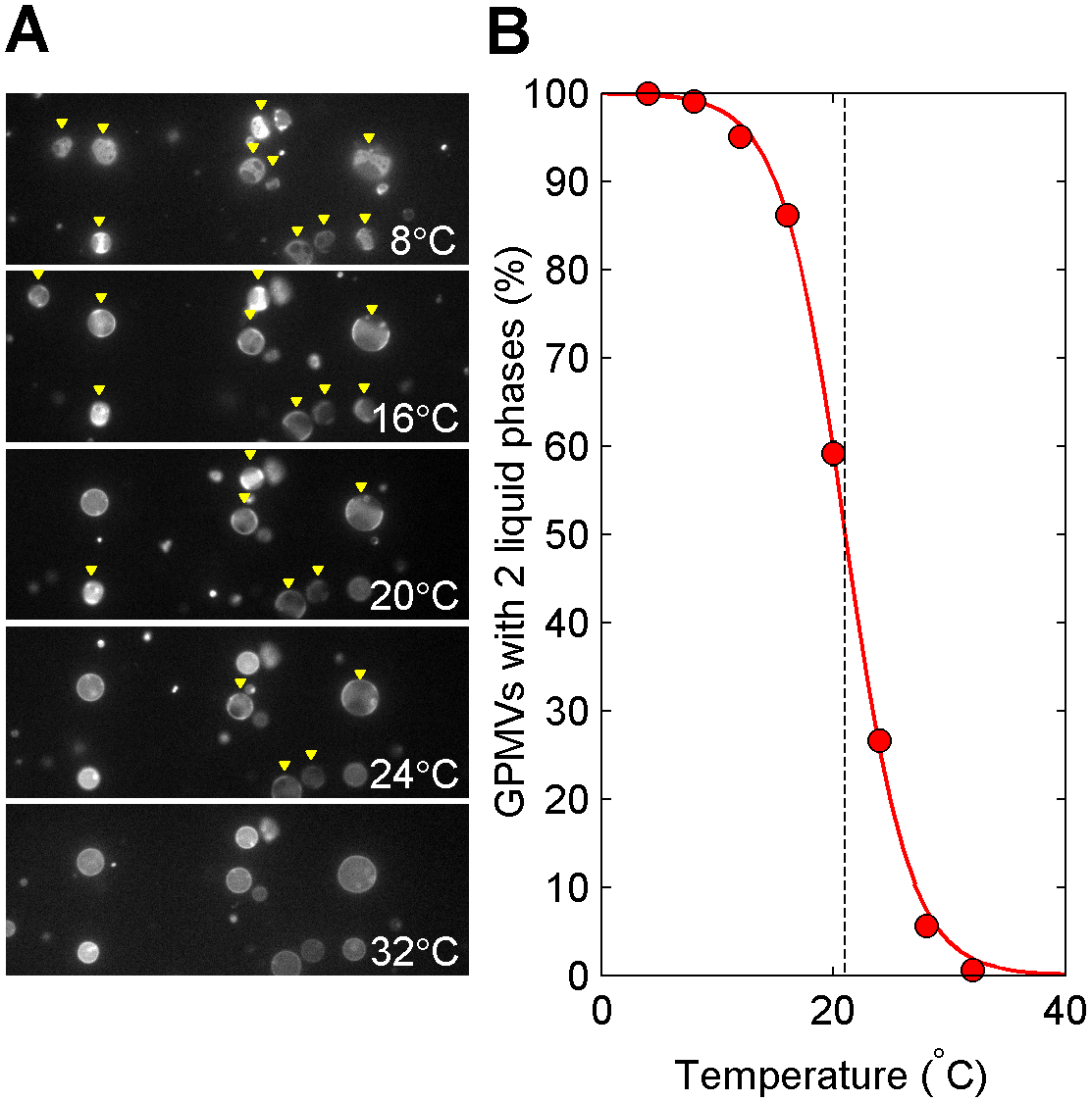
GPMVs from RBL-2H3 exhibit a broad distribution of miscibility transition temperatures. (A) A single field of GPMVs imaged at several distinct temperatures. Vesicles identified as containing coexisting liquid phases are marked with a yellow triangle. In this field, some vesicles contain coexisting phases and others are uniform at both 20° and 24°C. (B) Heterogeneity in the transition is quantified in a single GPMV preparation by measuring the fraction of vesicles containing two coexisting liquid phases as a function of temperature over many fields of vesicles like those shown in A. The average transition temperature is defined as the extrapolated temperature where 50% of vesicles are phase separated, which in this case is 21.0±0.2°C (dashed line). The width of the transition typically spans 10°C.

Giant plasma membrane vesicles (GPMVs) isolated from a dish of RBL-2H3 cells contain two coexisting liquid phases at low temperatures, a single liquid phase at elevated temperatures, and micron-sized critical composition fluctuations near the miscibility transition [10]. Typically, the miscibility transition occurs near or below room temperature when GPMVs are prepared using low concentrations of DTT and formaldehyde [8–10], as described in Materials and Methods. Even though there is a well-defined miscibility transition temperature for single vesicles, there is vesicle-to-vesicle heterogeneity which can span 5–15°C even when GPMVs are prepared from a flask of seemingly identical cells. This is demonstrated in Fig 1A, which shows vesicles imaged over a range of temperatures. At the lowest temperatures imaged (8°C and 16°C), all vesicles contain coexisting liquid phases, indicating that their transition temperature is positioned above the temperature of the microscope stage. When temperature is raised, a few vesicles in the field become uniform, indicating that the transition temperature of these vesicles falls somewhere between 16° and 20°C. As temperature is increased further, more vesicles become uniform until at a high temperature (32°C) all vesicles contain a single liquid phase. In order to measure the average transition temperature of the population of cells, the fraction of vesicles with two coexisting liquid phases is quantified as a function of temperature by imaging fields of GPMVs at several fixed temperatures (Fig 1B). These curves are fit with the sigmoid function of Eq 1 to determine the temperature where 50% of vesicles have passed through the transition, which we refer to as the average transition temperature of the sample (T_misc_). For the example, T_misc_ is 21.0 ± 0.2°C.

### Average transition temperatures vary with cell surface density

**Fig 2:**
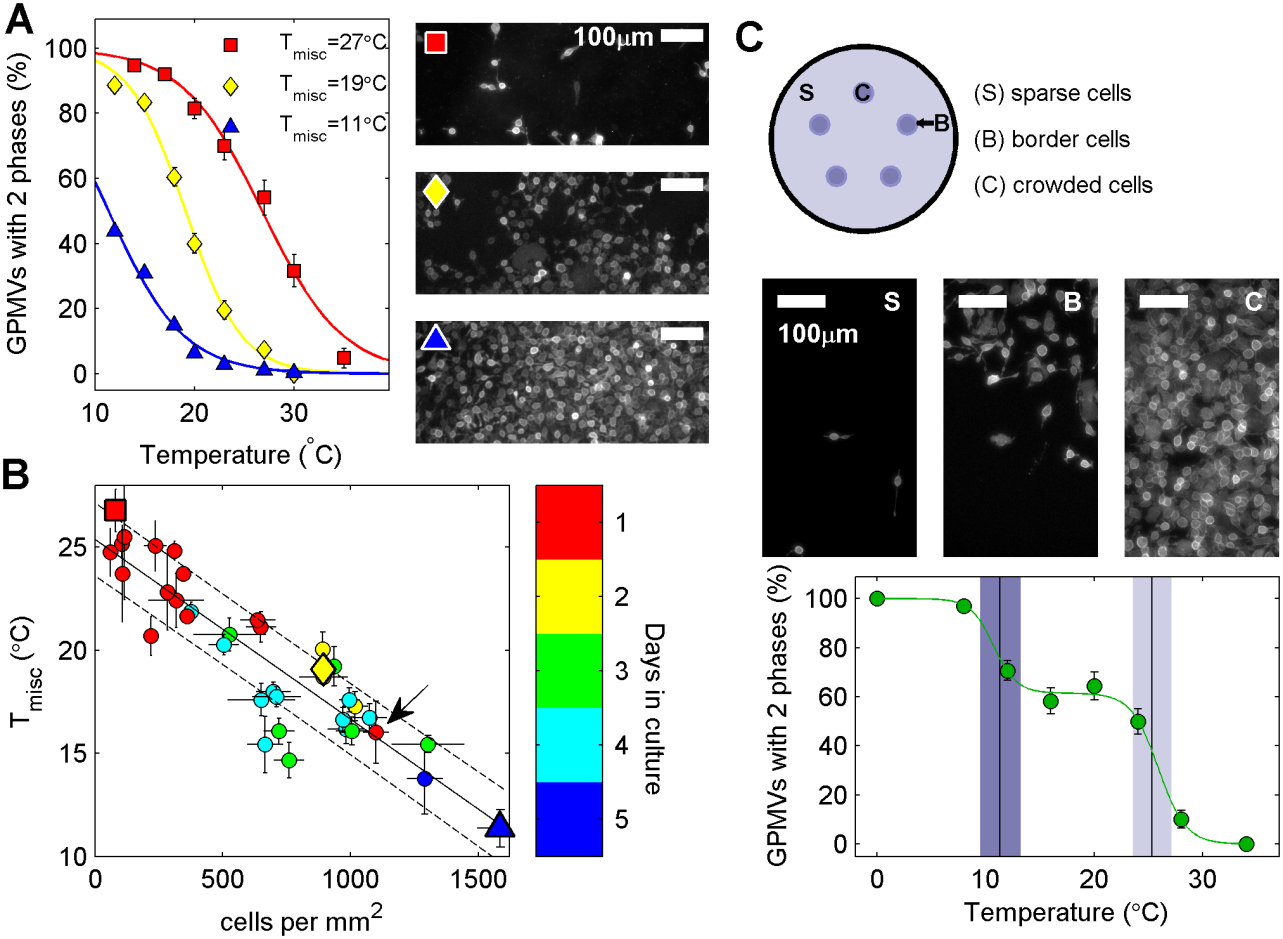
Transition temperatures are reduced in GPMVs isolated from more densely plated cells. (A) The percentage of GPMVs with coexisting liquid phases as a function of temperature varies significantly when GPMVs are prepared from cells plated at different densities. Images at right are representative fields of DiI-C_12_ labeled cells imaged prior to GPMV isolation for the indicated symbols. (B) Average GPMV transition temperature as a function of average cellular plating density. Color indicates the number of days between seeding and GPMV preparation, with an arrow pointing to a crowded sample that spent one day in culture. Average transition temperatures for representative samples shown in A are plotted with the same symbols in B. The solid line is a linear unweighted fit to the points and the dotted lines represent a standard deviation of the linear prediction. (C) GPMV transition temperatures are more heterogeneous when prepared from a dish of cells with large variations in local density. A dish of cells containing regions of high crowding and sparsely distributed cells was prepared as described in Methods. Images show representative regions of sparse, crowded, and border region cells. Points describing the percentage of phase separated vesicles as a function of temperature for GPMVs prepared from these cells are well described by a sum of two sigmoidal functions (green line). The inflection points correspond to the expected transition temperatures obtained using measured local cell densities and the trend line and confidence intervals shown in (B), as indicated by shaded boxes.

Average transition temperatures show large variation when comparing GPMVs isolated from cells plated at different densities. GPMVs prepared from sparsely plated cells contain coexisting phases to much higher temperatures than those prepared from densly plated cells. For the examples shown in Fig 2A, T_misc_ varies by more than 15°C between the sparsely plated and densly plated samples. Cell surface density was quantified by counting cells in DiI-C_12_ labeled flasks immediately prior to GPMV preparation as described in Materials and Methods. Fig 2B summarizes the results of 33 distinct measurements of plating density and GPMV T_misc_. We observe a robust linear trend with a negative slope between these two quantities over nearly 2 orders of magnitude in surface density and 15°C in T_misc_.

Fig 2B also notes the number of days each plate of cells remained in culture prior to GPMV preparation. Since RBL-2H3 cells double roughly every 18–24 hours, cells cultured longer tend to contain a higher density of cells. There is an exeption to this trend in Fig 2B indicated with an arrow. In this case, T_misc_ is well predicted by cell density alone. This suggests that density and not number of days in culture determines GPMV transition temperatures. To further test the dependence of GPMV transition temperatures on local cell density, we prepared dishes of cells with regions of high local crowding surrounded by sparsly distributed cells (Fig 2C). The population of GPMVs prepared from these samples undergo the miscibility transition over a much broader temperature range than samples prepared at uniform average density. For the example shown in Fig 2C, we can detect two distinct average GPMV transition temperatures by fitting the plot of % phase seperated vs. temperature to a sum of two sigmoidal functions from Eq 1. In this case the higher transition temperature is 26±1°C, which is in good agreement with temperatures observed in uniformly plated cells at a similar cell density (2–10 cells/mm^2^). The lower average transition temperature is 11±1°C, and is also in good agreement with values expected for densly plated uniform samples (1000–2000 cells/mm^2^). This provides further evidence that GPMV transition temperatures are determined by local and not average cell density, and that vesicle-to-vesicle heterogeniety in GPMV transition temperatures is likely a result of variation in the local density surrounding individual cells.

In many laboratory cell lines, including RBL-2H3s, growth rates gradually slow as cells become confluent due to contact inhibition [28,29]. Several past studies have also shown that growth rates also slow when cells are plated such that most cells do not have local cell contacts [30,31]. We have not observed a similar lowering of transtion temperatures for GPMVs isolated from the most sparsely distributed cells, suggesting that growth rates and plasma membrane transition temperatures are not directly linked. It is possible that instead cells use membrane physical properties to sense or communicate the presence of local cellular contacts, as is suggested by a recent study [32].

### Transition temperautres are reduced in serum starved cells

**Fig 3:**
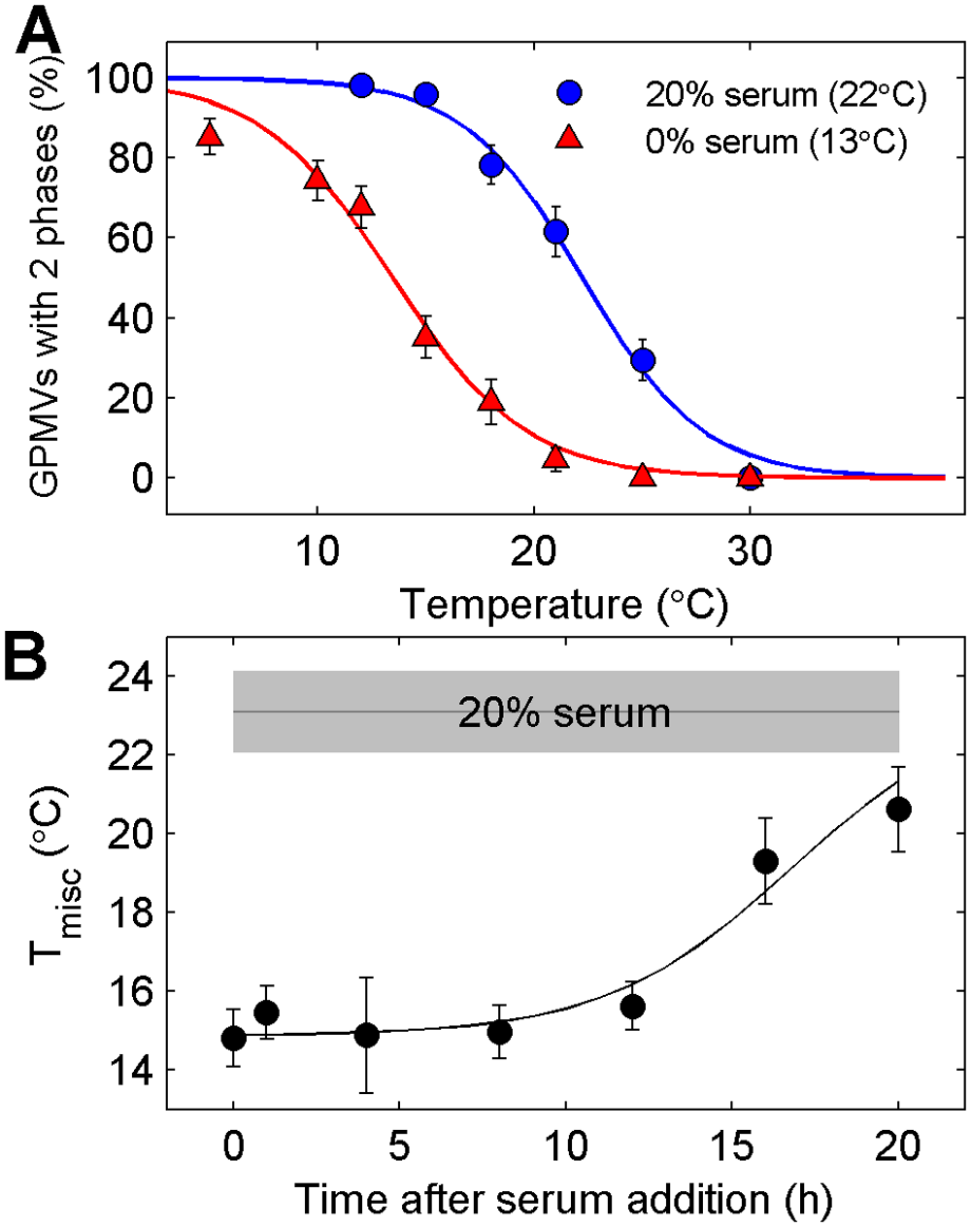
Transition temperatures are reduced in cells with arrested growth due to serum starvation. (A) Transition temperatures are suppressed in GPMVs isolated from cells incubated overnight in serum free medium when compared to GPMVs isolated from cells incubated in normal (20%) serum. (B) Elevated transition temperatures are restored in GPMVs isolated from serum starved cells in which serum has been restored for >15h. Points are a weighted average over three distinct measurements. Line is drawn to guide the eye and is not a fit to any theory.

Cellular growth is also arrested through removal of serum from growth media. We find that GPMV transition temperatures are dramatically reduced when cells are starved of serum overnight (Fig 3), even when cells have the same surface density prior to GPMV preparation. For the example shown in Fig 3A, we observe a 9°C downward shift in transition temperature. We find an average transition temperature for serum starved cells to be 8.5°C lower than GPMVs from control cells grown in complete media, with a standard deviation of 1.8°C over 6 separate experiments.

In order to determine if elevated transition temperatures in the presence of serum is due to the direct impact of a serum component or components, we examined how T_misc_ varied as a function of time after adding back 20% serum to serum starved cells (Fig 3B). Transition temperatures remain low in GPMVs prepared from cells at short times (<12h) after serum add-back, indicating that a serum component is not itself responsible for elevated transition temperatures. T_misc_ is increased after 16 or 20h incubation with media containing 20% serum. This time-scale is on the order of the doubling time of RBL-2H3 cells in this serum condition, suggesting that T_misc_ is elevated due to some slower metabolic process, possibly related to cell growth and division.

### Smaller changes in T_misc_ are observed within the cell cycle

Another possible source of heterogeneity is that GPMVs produced from cells at different stages of growth and division produce vesicles with different transition temperatures. To explore this hypothesis, we isolated GPMVs from collections of cells that were synchronized in their cell cycle through a double thymidine block, which arrests cells at the cell cycle checkpoint between G1 and S phases [25]. After release from the block, cells progress synchronously through the cell cycle through S, G2, M, and G1 phases. Synchronicity and cell cycle position was verified through DAPI staining of DNA in cells chemically fixed at the same time-points (S1 Fig).

**Fig 4:**
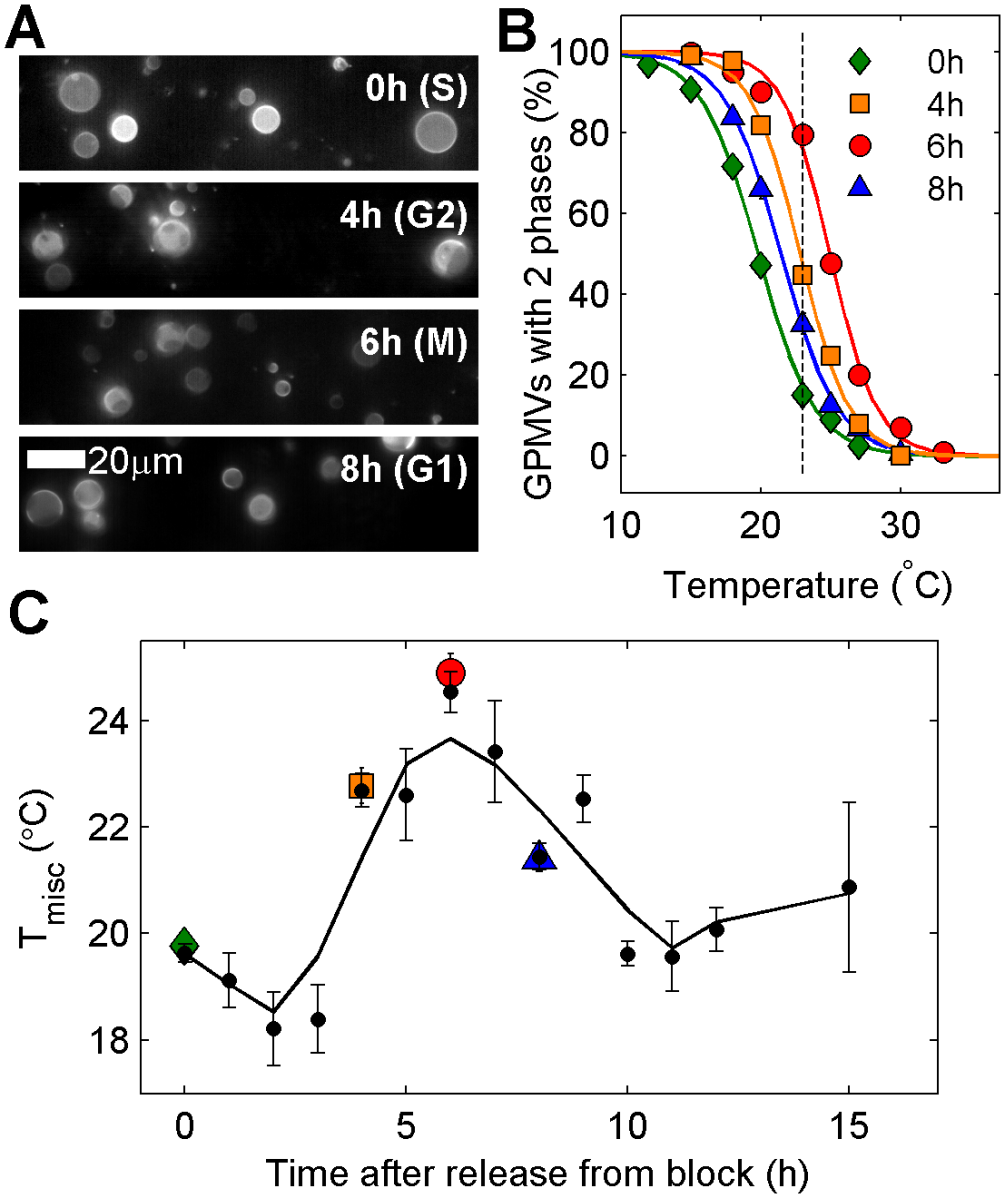
Transition temperatures vary systematically as RBL-2H3 cells synchronized to the G1/S boundary progress through the cell cycle. (A) Representative fields of GPMVs prepared at the indicated time-points after release from a double thymidine block and imaged at 23°C. GPMVs isolated immediately after release from block (top) are mostly in a single phase whereas GPMVs isolated at later time-points often contain two phases in coexistence. (B) The fraction of phase separated GPMVs as a function of temperature for GPMVs isolated at the indicated times after release from block. These curves are used to quantify the average transition temperature (T_misc_). The dashed line is drawn at 23°C for comparison to A. (C) T_misc_ as a function of time after release from the double thymidine block. Larger colored symbols represent values obtained in B and black symbols include data from 4 separate trials. The black line is smoothed from black points using a lowess filter.

Transition temperatures are systematically higher in GPMVs isolated from cells synchronized in G2 or M phases when compared to those measured in GPMVs isolated from cells in either G1 or S phases. This is seen qualitatively in Fig 4A, which shows representative fields of vesicles isolated at different time-points after release from block and imaged at fixed temperature (23°C). Curves representing the fraction of phase separated vesicles as a function of temperature are systematically shifted for GPMVs prepared from cells released at different times after release from block (Fig 4B). We note that there is still significant heterogeneity in transition temperature between vesicles, spanning roughly 10°C. This indicates that synchronizing cells does not lead to more homogeneous membrane composition by this measure. The trends in transition temperatures seen in a single experiment are reproducible over multiple measurements, as seen in Fig 4C where we show results from GPMVs isolated from cells at 1h increments after release from block. Here again it can be seen that T_misc_ is low soon after release from block when cells enter S phase, then increases prior to and during cell division (G2 and M phases). T_misc_ lowers again as cells enter G1 phase. It is possible that some lowering of T_misc_ can be attributed to increased average surface density following cell division. Overall, we find a 4–6°C shift in average transition temperatures over a complete cell cycle.

### Transition temperatures are reduced in GPMVs isolated from apoptotic cells

**Fig 5:**
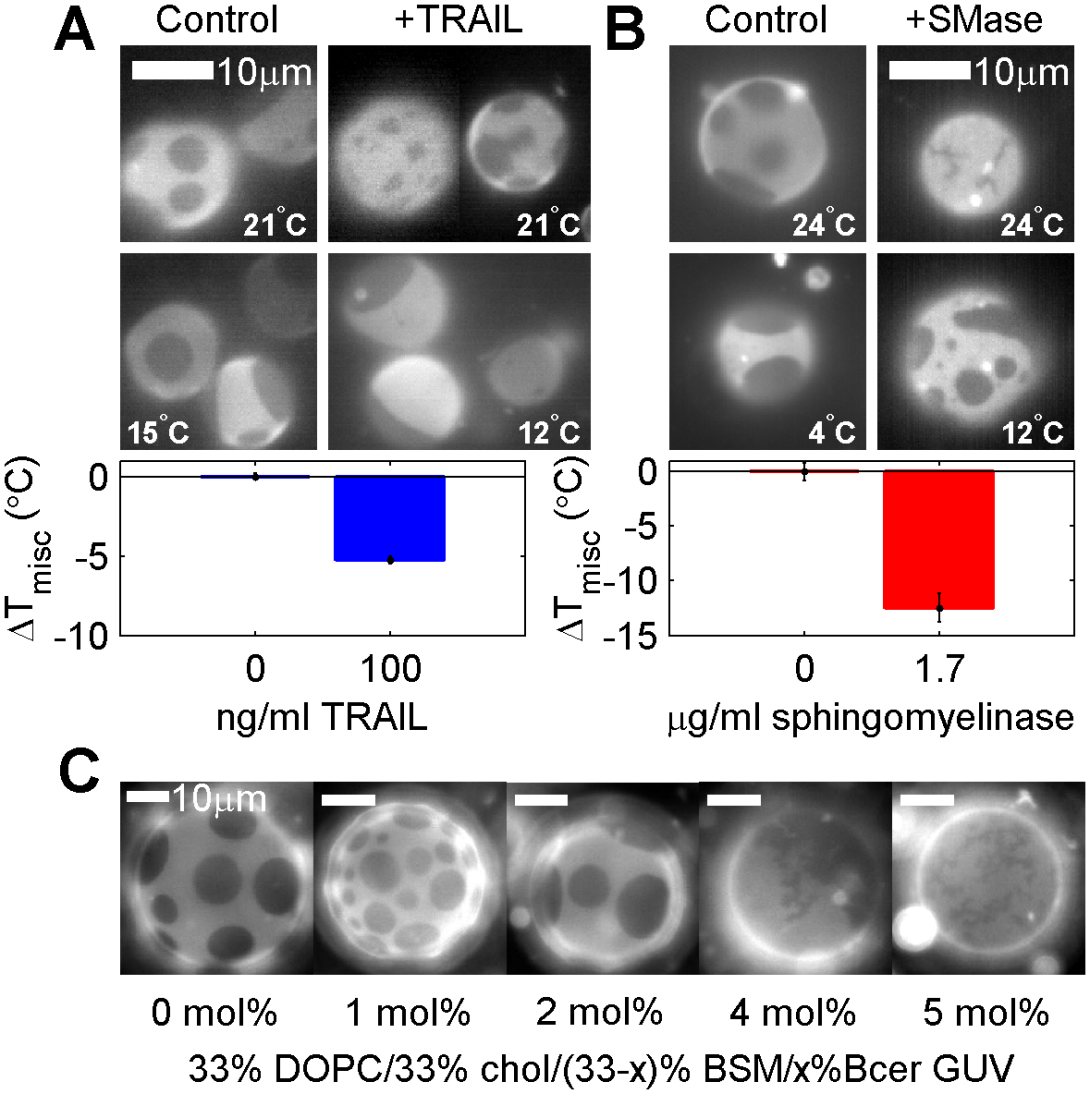
T_misc_ is reduced in GPMVs from apoptotic cells. (A) GPMVs isolated from RBL-2H3 cells pretreated with 100ng/ml TRAIL for 30min have lower transition temperatures than GPMVs isolated from untreated cells. At elevated temperatures, some TRAIL treated GPMVs contain non-circular domains. (B) GPMVs isolated from RBL-2H3 cells pretreated with purified sphingomyelinase (SMase) contain rigid and elongated gel domains at elevated temperature and more rigid liquid-like domains at lower temperature. GPMVs from sphingomyelinase treated cells have lower T_misc_ than control GPMVs, where T_misc_ is defined as the onset of the liquid appearing domains. (C) Elongated gel domains are also observed in GUVs containing purified DOPC, BSM, and Chol when 4–5 mol% of BSM is replaced by Brain ceramide. Images were acquired at 25°C.

There are numerous documented modifications of plasma membrane lipids that occur as cells undergo apoptosis, including conversion of sphingomyelin into ceramide and translocation of PS lipids to the extracellular leaflet. Fig 5 indicates that GPMVs isolated from apoptotic cells also have lower transition temperatures. Incubation with TNF-related apoptosis-inducing ligand (TRAIL) triggers apoptosis through binding and activation of several death receptors, followed by activation of sphingomyelinase which enzymatically converts plasma membrane sphingomyelin lipids into ceramide [33]. Cells incubated with TRAIL for 30min at 37°C produce GPMVs with transition temperatures that are well below those of control cells at the same plating density. Overall, phase separated domains in GPMVs isolated from TRAIL treated cells appear similar to controls at low temperature. At higher temperatures, some TRAIL treated vesicles contain a smaller surface fraction of ordered phase than control vesicles, and there is some evidence that ordered domains are more viscous, as indicated by the slow coalescence of domains (Fig 5A, 21°C).

Ceramides are produced directly without prior death receptor activation when cells are incubated with active sphingomyelinase [34]. Fig 5B shows results for vesicles prepared from cells treated with 1.7μg/ml of purified sphingomyelinase for 30 min at 37°C prior to GPMV isolation. An apparent gel phase is observed in some GPMVs at temperatures above the onset of the miscibility transition (24°C in Fig 5B), where gel domains are distinguished from liquid domains through their elongated appearance, rigid body rotation, and irregular boundaries that do not change with time [35]. At lower temperatures, a second liquid phase also appears, frequently obscuring a gel phase if present (12°C in Fig 5B). If T_misc_ is defined as the midpoint of the transition between a single liquid and two liquid phases, ignoring the presence of gel-like domains, then this T_misc_ is lower in sphingomyelinase vs. untreated vesicles. It should be noted that past work as shown that plasma membrane cholesterol levels are also reduced upon similar treatment with this enzyme [34], which could also contribute to these observations.

In general, these observations in GPMVs are consistant with past work in purified membranes, where replacement of sphingomyelin lipids with ceramide has been shown to destabilize liquid ordered domains and promote the formation of gel phase domains in membranes [17–19]. This is also demonstrated in Fig 5C for purified giant unilamellar vesicles (GUVs) of DOPC, cholesterol, Brain Sphingomyelin (BSM), and Brain ceramide (Bcer).

## Discussion

Here we have shown that GPMVs isolated from RBL-2H3 cells have miscibility phase transition temperatures that depend on cell cycle position and growth conditions. The highest transition temperatures are observed in GPMVs prepared from sparsely plated cells in the presence of serum, both conditions that support the proliferation of this cell type. In contrast, GPMV transition temperatures are lowest when isolated from densely plated or serum starved cells, conditions typically associated with arrested growth. We also observe more subtle but systematic changes in transition temperature from GPMVs isolated from cells that are synchronized in their cell cycle, as well as lower transition temperatures in GPMVs isolated from cells undergoing apoptosis.

A major goal of these measurements is to clarify the source of heterogeneity in miscibility transition temperatures between vesicles, which usually spans >10°C when GPMVs isolated from a plate of seemingly identical cells. This variation is likely not dominated by asynchrony of the cell cycle, since the 4°C shift in T_misc_ observed within the cell cycle in synchronized cells is small compared to the 10°C variation between vesicles in a typical unsynchronized sample, and the transition remains broad even in GPMVs isolated from synchronized cells. We conclude it is more likely that variation in transition temperature is dominated by the large surface density dependence on GPMV transition temperatures documented in Fig 2. Here we find that average GPMV transition temperatures vary by >10°C over conditions where the majority of cells go from having few distant neighbors to many closely packed neighbors. In a typical dish of cells, there can be a large variation in the local density surrounding individual cells. It is possible that this variation in local surface density could give rise to significant heterogeneity in transition temperatures of GPMVs isolated from these cells. This conclusion is further supported by our observations of a very broad transition in GPMVs from cells intentionally plated with large variations in local density as shown in Fig 2C.

In the closed GPMV systems studied here, variation in T_misc_ must arise due to variation in the lipid and/or protein composition of vesicles. It is most likely that this is reflective of changes in plasma membrane composition of the cells from which GPMVs are isolated. It is also possible that changing growth conditions could lead to differential sorting of plasma membrane components into GPMVs even if the plasma membrane composition itself is not changing significantly, possibly as a result of well characterized changes in the structure of cortical actin [36,37]. A number of past studies have identified how some plasma membrane components vary within the cell cycle [13]. In a recent study, the fractional content of arachidonic acid within phosphatidylcholine (PC) lipids in total lipid extracts increases as synchronized NIH-3T3 fibroblasts cycled through G1 phase [14]. In another recent study, certain lipids are found to be highly enriched in M vs. S phase lipid extracts in HeLa cells, including several ceramides, specific oxysterols, and a saturated either-linked phosphatidic acid (PA) lipids [15]. For the case of the RBL-2H3 cells used here, previous work has demonstrated that surface expression of the FcεRI receptor can be cell cycle dependent [38], and that in general S and M phase cells appear more differentiated [39]. It is expected that at least some plasma membrane components that vary within the cell cycle or under different growth conditions could give rise to significant changes in GPMV transition temperatures. Previous work has shown that GPMV transition temperature can be highly sensitive to the endogenous addition of lipophilic compounds such as general anesthetics or detergents [23,40].

**Fig 6:**
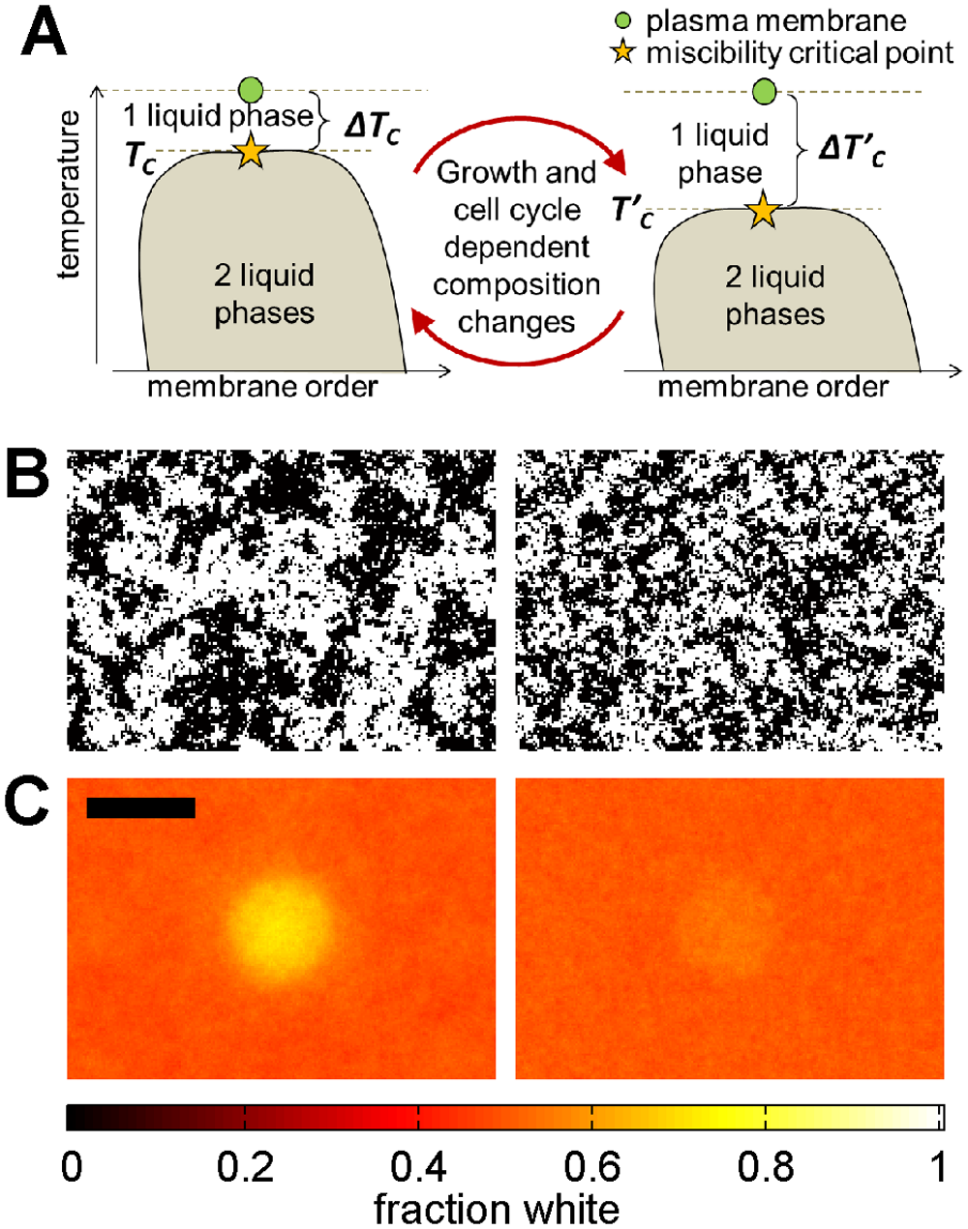
Predicted consequences of changing plasma membrane critical temperatures. (A) One model of lipid-mediated membrane heterogeneity postulates that intact cell plasma membranes are tuned to be slightly above a critical point under growth conditions, as represented by the schematic phase diagram shown. In this model, conditions that alter T_misc_ also alter ΔT_C_, or the difference between growth temperature and T_misc_. (B) Conditions that give rise to higher T_misc_ are predicted to also give rise to larger and more long-lived composition fluctuations at growth temperature (left) when compared to conditions with lower T_misc_ (right). (C) Membranes with higher T_misc_ are also predicted to be more susceptible to subtle perturbations, making it easier to stabilize large and long-lived structures, as evident in the time-averaged simulated images shown. Images in B and C have the same scale with scale-bar of 50 pixels and simulations were conducted at T = 1.05T_C_ (left images) and T = 1.2T_C_ (right images), where T_C_ is the critical temperature of the 2D Ising model. Methods used to generate the simulated images in B, C are described in Methods.

Based on this work alone, it is not possible to determine if the observed changes in GPMV transition temperatures are a cause or consequence of growth or cell cycle dependent functions. In light of our current results showing robust changes in transition temperatures within the cell cycle and under different growth conditions, it is tempting to speculate that cells are actively tuning plasma membrane composition in order to promote or inhibit the formation of lipid mediated heterogeneities in the membrane, such as those generally associated with lipid rafts [41,42]. We have previously proposed a critical fluctuation model of plasma membrane heterogeneity, in which composition fluctuations resembling low temperature membrane phases persist to physiological temperature because the plasma membrane is biologically tuned to a miscibility critical point [10,27]. Within this model, the size, magnitude, and lifetime of composition fluctuations depend on the temperature difference between growth conditions and the lower temperature miscibility transition of the membrane (Fig 6). In purified and isolated biological membranes, the unique physical properties of super-critical membranes have been used to stabilize large-scale domains in vesicles adhered to a supported membrane [24] or in membranes coupled to an actin cortex [43,44]. Theoretical studies predict more robust compartmentalization of membranes coupled to actin [27], greater sensitivity to components proximal to the plasma membrane [45], and stronger and longer-range lipid-mediated interactions between proteins [46] when membranes are poised closer to a miscibility critical point.

If GPMV miscibility transition temperatures are indeed representative of the intact plasma membranes from which they derive, then one possibility is that cells actively tune their plasma membrane composition in order to tune the physical properties of their membranes to accomplish some biological function. For example, it is possible that elevated plasma membrane transition temperatures in mitosis act to lower the energy cost of assembling proteins and lipids at and around the cleavage furrow, that depressed critical temperatures under low nutrient growth conditions act to inhibit energetically expensive ‘raft’ dependent signaling events, or that reduced critical temperatures early in apoptosis somehow facilitate interactions between death receptor complexes and downstream components. It is also possible that changes in the mixing properties of membrane lipids contribute to previously documented differential responses to stimuli in some cell types under different growth conditions or cell cycle stages [47–49]. Future work is required to explore if these or other possible consequences of changing GPMV transition temperatures are found intact and living cell membranes at growth temperature.

## Supporting Information

S1 Fig DAPI staining of RBL-2H3 cell nuclei indicate cells remain synchronized after a double thymidine block. RBL-2H3 cells were chemically fixed in 4% paraformaldehyde and 0.1% glutaraldehyde at the time points indicated, then stained with DAPI, which labels DNA, prior to imaging. GPMV preparation requires incubation in activated buffer for 60min, therefore cells were fixed 30min after the typical onset of GPMV preparation in the measurements described in the main text. DNA begins to replicate at the start of S phase, and nuclei are heterogeneous in size when cells are fixed soon after release from block. Nuclei are uniformly large after 4h of release from block, consistent with cells being in G2 phase. By 6h after release from block, a significant fraction of cells are undergoing active division, and DAPI labels structured chromosomes that are dividing symmetrically. This is indicative of cells in the M phase. Cells are more numerous when fixed at 8h after release from block and nuclei are smaller, indicating that most cells have undergone cell division and have entered the G1 phase. Red circles have the same diameter in all images shown.
(TIF)
