# Supplementary figures and images for: Growth Conditions and Cell Cycle Phase Modulate Phase Transition Temperatures in RBL-2H3 Derived Plasma Membrane Vesicles
Source: PLoS One. 2015 Sep 14;10(9):e0137741. doi: 10.1371/journal.pone.0137741 (PMC4569273; doi:10.1371/journal.pone.0137741)

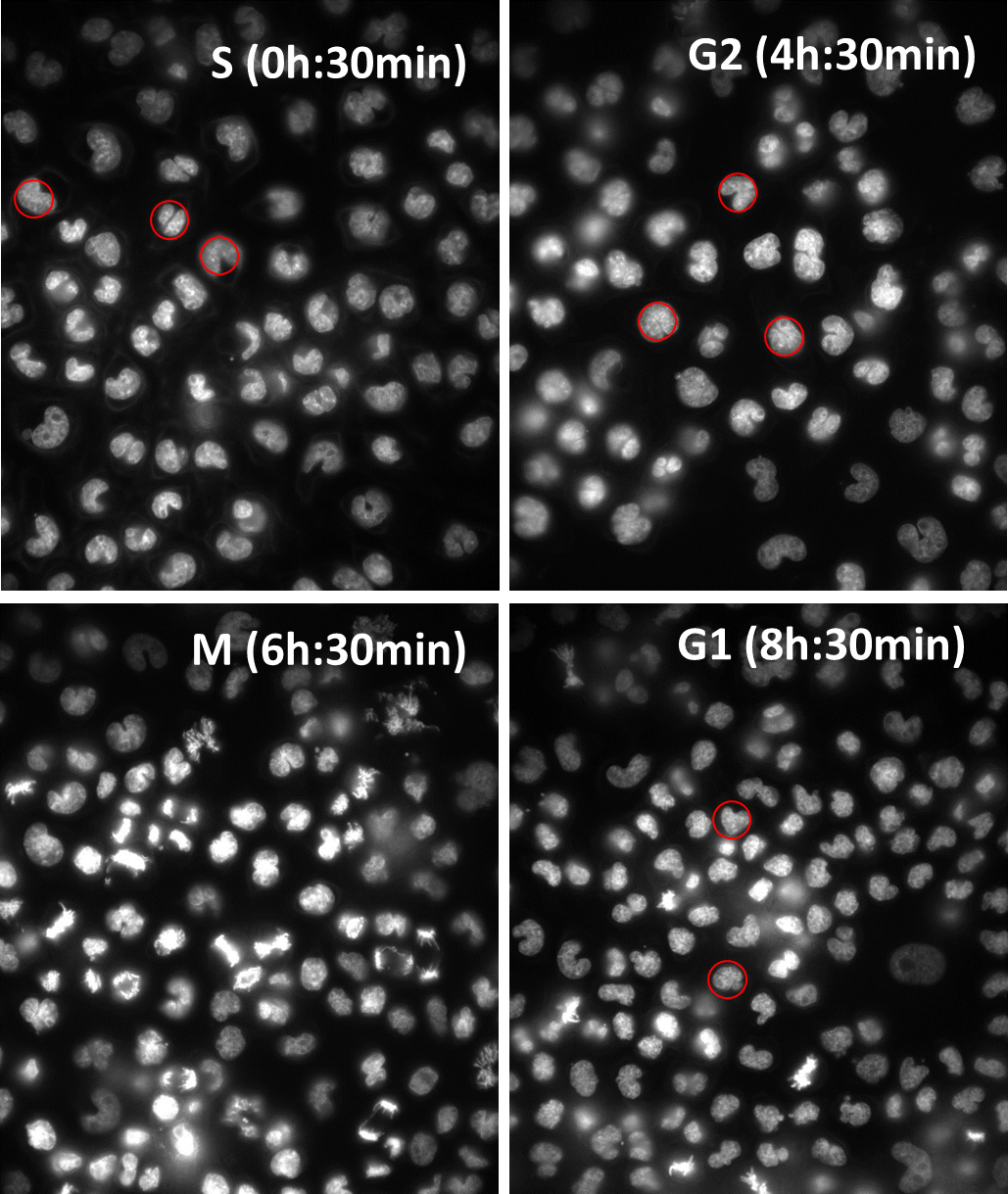

Supplement: S1 Fig — RBL-2H3 cells were chemically fixed in 4% paraformaldehyde and 0.1% glutaraldehyde at the time points indicated, then stained with DAPI, which labels DNA, prior to imaging. GPMV preparation requires incubation in activated buffer for 60min, therefore cells were fixed 30min after the typical onset of GPMV preparation in the measurements described in the main text. DNA begins to replicate at the start of S phase, and nuclei are heterogeneous in size when cells are fixed soon after release from block. Nuclei are uniformly large after 4h of release from block, consistent with cells being in G2 phase. By 6h after release from block, a significant fraction of cells are undergoing active division, and DAPI labels structured chromosomes that are dividing symmetrically. This is indicative of cells in the M phase. Cells are more numerous when fixed at 8h after release from block and nuclei are smaller, indicating that most cells have undergone cell division and have entered the G1 phase. Red circles have the same diameter in all images shown. (TIF) [file pone.0137741.s001.tif]
